# Supplementary material for: Creating Acceptable Tablets 3D (CAT 3D): A Feasibility Study to Evaluate the Acceptability of 3D Printed Tablets in Children and Young People
Source: Pharmaceutics. 2022 Feb 25;14(3):516. doi: 10.3390/pharmaceutics14030516 (PMC8954179; doi:10.3390/pharmaceutics14030516)
Supplement: Supplementary file 1 [file pharmaceutics-14-00516-s001.zip › pharmaceutics-1587269-supplementary.pdf]

Research paper

Creating Acceptable Tablets 3D (CAT 3D): A feasibility study to evaluate the mouthfeel of 3D printed tablets in children and young people.

Supplementary Data

Louise Bracken[1], Rober Habashy[2], Emma McDonough[1], Fiona Wilson[3], Joanne Shakeshaft[1], Udeme Ohia[3], Tamar Garcia-Sorribes[2], Mohamed A Alhnan[4,*], Matthew Peak[1, 3] **

[1] Paediatric Medicines Research Unit, Alder Hey Children's NHS Foundation Trust, Liverpool, L12 2AP, UK.

[2] School of Medicine and Biomedical Science, University of Central Lancashire, Preston, PR1 2HE, UK.

[3] NIHR Alder Hey Clinical Research Facility, Alder Hey Children's NHS Foundation Trust, Liverpool, L12 2AP, UK.

[4] Centre for Pharmaceutical Medicine Research, School of Cancer & Pharmaceutical Science, King's College, London, SE1 9NH, UK.

*Corresponding author:
Dr Mohamed A Alhnan
Centre for Pharmaceutical Medicine Research
Institute of Pharmaceutical Sciences
King's College London
150 Stamford Street
London SE1 9NH. Tel.: +44 (0)20 7848 726
Email: Alhnan@kcl.ac.uk

**Corresponding author:
Professor Matthew Peak
Paediatric Medicines Research Unit
Alder Hey Children's NHS Foundation Trust
Liverpool
L12 2AP.
Email: matthew.peak@alderhey.nhs.uk

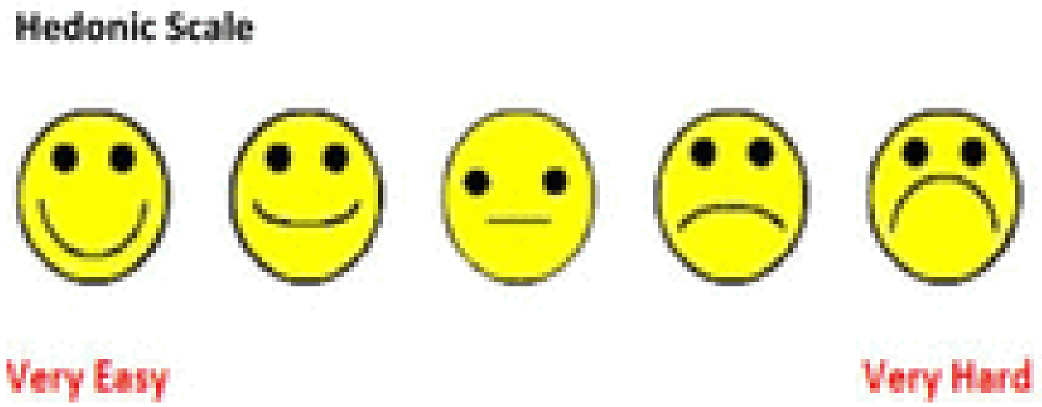

Figure S1 Hedonic scale on which participants were asked to rate 3D printed tablets.

Table S1 Summary of heavy metal assay of 3D printed tablets.

Element	Cd	Pb	As	Hg	Co	V	Ni	Cu
**Limit ppm**	0.5	0.5	1.5	3	5	10	20	300
**Ammount (ppm)**	0.33±0.21	0.68±0.56	0.72±0.27	0.03±0.023	0.24±0.07	0	0.16±0.08	1.12±0.35
**Amount in 10-mm tablet in µg**	0.11	0.23	0.25	0.01	0.08	0.00	0.05	0.40
**Amount in 8-mm tablet in µg**	0.06	0.12	0.12	0.00	0.04	0.00	0.03	0.20
**Amount in 6-mm tablet in µg**	0.03	0.06	0.06	0.00	0.02	0.00	0.01	0.10
